# A demographic survey of unwanted horses in Ireland 2005-2010

**DOI:** 10.1186/2046-0481-65-3

**Published:** 2012-03-02

**Authors:** DP Leadon, Dylan O'Toole, Vivienne E Duggan

**Affiliations:** 1 Irish Equine Centre, Johnstown, Naas, Co Kildare, Ireland; 2 School of Veterinary Medicine, University College Dublin, Belfield, Dublin 4, Ireland

**Keywords:** Equine, Unwanted horse, Ireland

## Abstract

**Background:**

The Irish Horse Industry expanded during the Celtic Tiger boom years, then contracted in the current economic recession. High value horses were traditionally controlled through sale at public auction, private sales and sales to dealers; these are now also being reduced by decreases in production (> 40%), and increases in retirement, re-homing, euthanasia and disposal through Category 2 plants and abattoirs. The absence or banning of horse abattoirs has been shown to have very significant welfare social and economic consequences in the USA. This study described the currently available data on the demographics of unwanted horses in Ireland from 2005 to 2010.

**Results:**

The majority of horses euthanised by practicing veterinarians are destroyed on medical grounds but the number euthanised at the request of welfare groups and the state, as well as welfare related calls and the number of horses involved in these calls and subsequent visits is increasing reflecting the increasing involvement of the veterinary profession in equine welfare. Welfare groups have limited resources and do not have a tradition of recording data, but they too have reported increasing calls, visits and numbers of horses per visit. Welfare groups provide significant service to equine welfare and the community. Local Authorities report similar trends. Over 300 horses were found dead or required immediate or subsequent euthanasia following welfare group and local authority visits in 2010, which is of national concern. The majority of local authority interfaces with unwanted horses are with urban (60%) rather than rural (40%) horses. Mortality figures are poor indicators of non-fatal neglect. More horses were admitted into the care of local authorities than welfare groups, reflecting significant state and taxpayer investment in the control of low value horses. Category 2 plants and abattoirs represent a significant state investment in licensing and control in the national interest. Abattoirs provide an increasingly important and essential service for the disposal of unwanted horses. Despite the increase in unwanted horses, Ireland is a minority contributor to the EU slaughter total.

**Conclusions:**

There is a need for annual demographic data compilation and review of the numbers of unwanted horses and ponies within the horse industry to assist policy makers and legislators.

## Background

Ireland's horse and pony population fell progressively throughout the mid twentieth century from 402,000 in 1949 to under 300,000 by 1955 and by 1962 to less than 200,000. The rate of decline decreased thereafter and from 98,000 in 1974 to 59,000 in 1989 until sport, leisure and tourism stimulated expansion [1]. Government figures of horse populations are acknowledged to be underestimates and it has been demonstrated [2] that the size of the equine population of Scotland and Northern England was more than three times the number recorded in the MAFF annual census. Although there is a legal requirement for all horses in Ireland to be identified and be in possession of a valid passport within six months of birth or by 31st December of the year of birth, it is generally recognised that there is poor compliance with this legislation, other than in the elite sectors.

Passports issued for Thoroughbreds and Sporthorses provide accurate data on these sectors of the horse population. Numbers of Thoroughbred broodmares in Ireland increased during the years of the economic boom from 11,950 in 1995 to 20,028 in 2008. In 2007, Ireland produced more Thoroughbred foals (12,633) than the combined total (11,233) for the UK (5,839) and France (5,394) [3]. Numbers of Thoroughbred racehorses in training increased from 8,028 in 2000 to 12,119 in 2008 [4]. The estimate for the Sporthorse population of Ireland in 2007 was 110,000 of which there were on average 27.5 sport/leisure horses per 1,000 people. This figure placed Ireland as the most horse-dense population of Europe. The Irish Sport Horse Studbook is the largest sport horse breed in Ireland, registering 7,440 foals in 2007. This represented around 70% of all studbook-registered sport horses and ponies in Ireland, making it the fourth largest sport horse studbook in Europe surpassed in numbers only by the Dutch Warmblood (KWPN), the Hannoverian and the Selle Francais [5].

Global recession which commenced in 2008 has meant that many horses in many countries have become unwanted. Unwanted horses have been defined in the USA as those that may be sick, injured or old, or be those that owners are no longer able to afford, fail to meet their owners' expectations or simply be those that their owners no longer know what to do with [6]. Industry bodies (the Irish Thoroughbred Breeders' Association--ITBA and Horse Sport Ireland--HSI) responded to the changed economic situation and to perceived overproduction by urging their members to practice responsible breeding and ownership. Five means of disposal of unwanted horses have been advocated by the ITBA and HSI; retirement, re-homing/re-training, euthanasia (by the veterinary profession) and disposal, through Category 2 plants/knackeries for rendering at the owners expense, or entry to the EU human food chain through submission for payment for carcass value, to licensed abattoirs[7] and [8].

Irish Welfare groups, which provide advice, re-homing and in some instances retraining or arrange for euthanasia, have reported increasing calls, to unwanted horses rising from 435 in 2008 to 2,014 in 2010 in one instance and some are in favour of an amnesty for slaughter while others are not [9]. Guidelines for the operation of equine sanctuaries within the State of California, the first of their type to be produced, have been published recently [10]. Guidelines for Equine Practitioners, Adoption Organisations and Horse Owners on Transitioning the Retired Racehorse produced by the American Association of Equine Practitioners [11] state that racehorses can be very useful in other careers after retirement, but that they require comprehensive (including veterinary) pre-retirement assessment and that the costs of housing retraining and re-homing retired racehorses can be considerable. Since 1995 Ireland's DAFF (Department of Agriculture, Fisheries and Food) has provided over €12 million to national animal welfare charity organizations and €1.210 million in 2010--(personal communication--Animal Health and Welfare Division DAFF).

There are no demographic surveys of the equine welfare groups in the EU. A Demographic study of USA based equine welfare groups characterized those organizations as being in existence for on average only six years, financially supported through donations and personal funds, dedicated to the care of only 10 to 20 horses on a property of just over 30 acres, and reliant on volunteers for help. Relinquished horses in the USA consisted of mostly light horse breeds (79.3%) with Thoroughbreds and Quarter Horses as the most represented breeds. The age of relinquished horses ranged from 3 d to 42 years old (12.4 ± 0.5 year). About half of the horses entered in the US survey were considered unhealthy due to illness, injury, lameness, or low body condition. For every four horses relinquished to these US non-profit organisations, only three horses were adopted or sold between 2006 and 2009, and many organizations had refused to accept additional horses for lack of resources. Funding was the greatest challenge to continued operation of non-profit equine organizations. Financial hardship, physical inability or lack of time to care for the horses by owners were the most common reasons for relinquishment, followed by seizure through law enforcement agencies for alleged neglect or abuse. The overall distribution of unwanted equids was comparable with the general US population [12].

Local Authorities also have a responsibility for unwanted horses in Ireland and between 2007 and 2010 DAFF paid more than €5.25 million to local authorities for costs relating to horse seizures, under the Control of Horses Act of 1996. Total payments since 2007, have increased annually by about €400,000 since 2007 and in 2010 alone, the Department of Agriculture, Fisheries and Food paid more than €2.5 million to reimburse local authorities for expenses relating to the seizure and recovery of abandoned horses and ponies.

Unwanted horses can be disposed of in Ireland through Category 2 plants, formerly known as Knackeries and through licensed abattoirs. Similar systems operate in other countries and for example 7,933 equidae were slaughtered in England, Scotland and Wales in 2010 [13]. The Equine Welfare Liaison Group of the Farm Animal Welfare Advisory Council (FAWAC) has stated that where a horse presents with severe welfare problems, where the owner cannot be identified and the horse can no longer fulfill the purpose for which it was bred, then it should be humanely disposed of. Domestic slaughter of horses in the United States, however, was banned in 2007. In 2009 Congress directed the Government Accountability Office [14] to examine horse welfare in the aftermath of the slaughter ban. The subsequent report found that horse exports from the US to Canada and Mexico had increased with nearly the same number of horses processed in those countries (138,000 in 2010) as were processed in the US before the ban was put in place. The economic recession has contributed to a sharp increase in the number of unwanted horses throughout the US, with estimates totaling 100,000 horses per year.

Although horses are specifically bred for horsemeat in many countries and large numbers of horses are processed for human consumption in other EU states, neither deliberate breeding of horses for horsemeat nor human consumption of horsemeat have been traditionally practiced in Ireland.

In Ireland as in the USA it is clear that unwanted horses have become a social and economic problem [15] and that the current understanding of the extent and magnitude of the problem is fragmentary and emotive. Industry bodies have called for clarification on the extent of the problem.

This survey aimed to quantify the numbers of horses born, sold, retired, re-homed, euthanised, or sent to Category 2 plants or abattoirs by horse owners in Ireland from 2005-2010. It aimed to describe the veterinary involvement in euthanasia and calls to the profession to visit unwanted horses at the request of welfare groups and local authorities. It also aimed to document the numbers of calls, group size, visits, horses actually present and numbers found dead or moribund by welfare groups and Local Authorities and on numbers of horses seized, released and sent for slaughter. Central Government data was sought on national involvement in unwanted horses by Local Authorities and the number of horses sent for entry into the human food chain through licensed abattoirs. The study sought independent corroboration of the central data. FAO data was sought to place the Irish data into the EU context and in so doing the study aimed to provide a template for further studies in Ireland and in other EU countries.

The study framework aimed to provide a template so that an annual review of the demographics and magnitude of the unwanted horse population will be available to industry stakeholders, the veterinary profession, welfare groups, to Local Authorities and national Government to assist with policy making and if required, legislation

## Methods

### Surveys

A link to an online survey facility (Survey Monkey) was circulated to: horse owners, private veterinary practitioners, equine welfare organizations, local authority veterinarians, managers of Category 2 plants and to managers of abattoirs slaughtering horses for human consumption,

1,100 members of the Irish Thoroughbred Breeders' Association (ITBA) were sent the survey by email through the offices of the ITBA and Horse Sport Ireland (HSI) also sent the same survey to the Affiliate Secretaries of its 19 constituent organizations who have a combined total of 20,364 members. Data on foal production was obtained from the ITBA and HSI. One thousand two hundred and twenty two private veterinary practitioners (PVPs) that were members of Veterinary Ireland, the national representative body for veterinarians, and a further 873 non-member veterinary practitioners received an e-mail survey. A list of equine welfare organisations and sanctuaries (including the Donkey Sanctuary) was compiled through a web based search and email addresses sought. The survey was circulated online to these organizations. A survey was circulated online to 44 Local Authority Chief Veterinary Officers through Veterinary Ireland. A list of Category 2 plants (38) and abattoirs (five in total: B & F Meats in Kilkenny, Ballon Meats in Co. Carlow, Shannonside Foods in Co. Kildare, Ashgrove Wholesale Ltd in Co Limerick and Ossory Meats in Co. Offaly) was compiled. Surveys were circulated online where email addresses were available. Otherwise, the survey was sent in paper form. The surveys were circulated with a cover letter explaining the purpose of the survey. A reminder was sent after 14 days and the surveys were closed 28 days after circulation.

All questions related to each of the years 2005 to 2010. Horse owners were asked how many horses they kept, sold (by public auction, private sale or to a dealer), retired, re-homed, had euthanised or disposed of via Category 2 plants or abattoirs. Members of the practicing veterinary profession were asked how many horses they euthanised for medical conditions or at the owner's request, or at the request of an Garda Siochana or the Local Authority. They were also asked how many visits they made to abandoned or neglected horses and how many horses were involved. Welfare groups and Local authority veterinarians were asked how many calls they received, and how many visits they made in response to these calls and how many horses were involved in each call or visit. They were asked for age, (< 5, 5-10, 10-15, 15-20 and > 20 years) breed/type(Pony, Horse, No breed, Sporthorse, Thoroughbred) and gender of horses they received calls about and they visited. They were asked how many horses they found dead on premises, and how many required immediate or subsequent euthanasia. Local authorities were asked how many horses they seized, released and euthanised. Managers of Category 2 plants and abattoirs were asked about the throughput of horses in each year and their type and age distribution. National Statistics on Local Authority involvement with unwanted horses and Category 2 plants and Abattoirs was obtained from the Department of Agriculture Food and the Marine (DAFM) and on national debt, from the Central Statistics Office. Data on mandatory Trichinella testing of equine carcasses from Irish abattoirs was obtained from the Microbiology Unit of the Irish Equine Centre. Data on horses slaughtered in the EU was obtained from the FAO.

### Data management and analysis

Responses were collated by the authors using the survey tool and the results were exported to Microsoft Excel (Microsoft Corporation, Redmond, WA, USA) for analysis.

## Results

Totals were calculated for the number of respondents from each category surveyed (Table 1) and the response rate varied from 1.3% for owners to 25% for welfare groups.

**Table 1:** Total Responses by category surveyed (excluding Central Government)

	Total surveyed	Responses
Owners	21,464	287 (1.3%)

PVPs	2,095	74 (3.5%)

Welfare groups	40	10 (25%)

Local Authorities	44	7 (16%)

Category 2 Plants	38	6 (16%)

Abattoirs	5	1 (20%)

**Figure 1:**
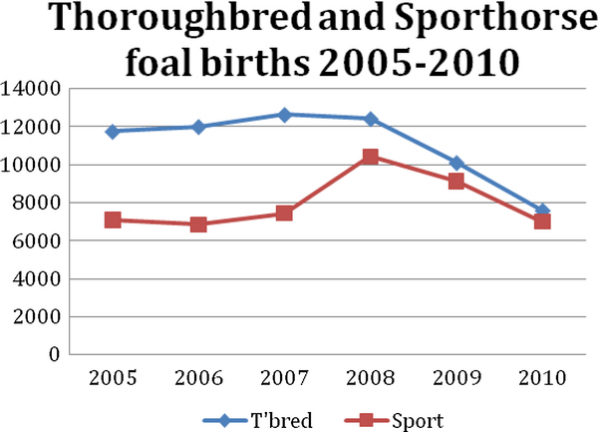
**Thoroughbred and Sporthorse foal births 2005-2010**.

Thoroughbred and sport horse births rose from 11,748 and 7,049 respectively in 2005 to peaks of 12,633 for thoroughbreds in 2007 and 10,424 in 2008 for Sporthorses, falling to 7,588 and 7,004 for respectively both breeds in 2010. This represented a 40% reduction in thoroughbred foal production and a 37% reduction in Sporthorse production (Figure 1)

### Owners--members of the ITBA and HSI affiliates

The 287 Horse Owners (Table 1) kept 2,536 horses in 2005, of which a total of 602 (23.7%) were sold, either at public auction, privately or to dealers (Table 2). A total of 125 (5%) were retired or re-homed and 48 (1.9%) were euthanised. A total of 34 (1.3%) were sent to Category 2 plants or abattoirs. They kept 2,738 horses in 2010 of which a total of 638(23.3%) were sold, either at public auction, privately or to dealers (Table 2). A total of 198 (7.2%) were retired or re-homed and 61 (2.2%) were euthanised. A total of 62 (2.3%) were sent to Category 2 plants or abattoirs. The total number of horses in their individual care ranged from 1 to 200 horses (mean 10 ± 24; median 3-5).

**Table 2:** Total horses kept and number of horses sold and disposed of, arranged by category of sale and disposal

	2005	2006	2007	2008	2009	2010
Horses kept	2536	2637	2757	2759	2748	2738

Public auction	404 (15.9%)	351 (13.3%)	407 (14.8%)	399 (14.5%)	411 (15%)	406 (14.8%)

Private sale	139 (5.5%)	147 (5.6%)	138 (5%)	139 (5%)	166 (6%)	134 (4.9%)

Sold to Dealer	59 (2.3%)	56 (2.1%)	71 (2.6%)	87 (3.2%)	83 (3%)	98 (3.6%)

Retired	70 (2.8%)	66 (2.5%)	64 (2.3%)	70 (2.5%)	69 (2.5%)	82 (3%)

Re-homed	55 (2.2%)	44 (1.7%)	51 (1.8%)	62 (2.2%)	81 (2.3%)	116 (4.2%)

Euthanasia	48 (1.9%)	34 (1.3%)	40 (1.5%)	47 (1.7%)	64 (2.3%)	61 (2.2%)

Category 2 plant	26 (1%)	18 (0.7%)	27 (1%)	46 (1.7%)	32 (1.2%)	32 (1.2%)

Abattoir	8 (0.3%)	7 (0.3%)	9 (0.3%)	14 (0.5%)	16 (0.6%)	30 (1.1%)

### Practicing veterinary profession

#### Euthanasia

In 2005, 159 of the 174 horses (91.3%) euthanised by 69 veterinary practitioners were destroyed on medical grounds and the remaining 15 horses (8.7%) were euthanised at the request of Owners, Gardai or Local Authorities (Table 3). In 2010, 79 practitioners reported that 209 of the 237 horses (88.2%) euthanised were on medical grounds and a total of 28 (11.8%) were at the request of owners, the Gardai or Local Authorities.

**Table 3:** Number of horses euthanised by the practicing veterinary profession on medical grounds, or at the request of owners, the Gardai and local authorities

	2005	2006	2007	2008	2009	2010
Vets	69	70	75	77	77	79

Total euthanised	174	190	203	230	229	237

Medical Conditions	159 (91.3%)	180 (94.7%)	192 (94.6%)	215 (93.5%)	199 (86.9%)	209 (88.2%)

Owner request	10 (5.7%)	7 (3.9%)	8 (4%)	10 (4.3%)	24 (10.5%)	13 (5.5%)

Gardai or Local Authority request	5 (3%)	3 (1.4%)	3 (1.4%)	5 (2.2%)	6 (2.6%)	15 (6.3%)

#### Visits

Sixty nine veterinary practices made a total of 18 visits to unwanted horses at the request of welfare groups, the Gardai and Local Authorities in 2005 and 15 of these visits (83.3%) were to groups of 1 to 5 horses (Table 4). Seventy nine practices made a total of 31 of these visits in 2010, 21 of which were to groups of 1-5 horses (67.7%), two to 5-10, one to 20 to 50 and there were two visits to > 100 horses.

**Table 4:** Number of visits made by veterinary practitioners to groups of abandoned or neglected horses at the request of local authorities, welfare groups or an Garda Siochana and the number of horses seen on these visits

	2005	2006	2007	2008	2009	2010
TOTAL Visits	18	17	18	19	26	31

No of Horses per visit						

1-5	15	13	14	16	20	21

5-10		1	2	1	2	5

10-20	2	2	1	1	3	2

20-50	1	1	1			1

50-100						

> 100				1	1	2

### Welfare groups

#### Calls

Three of the ten charities (Table 1) reported calls relating to unwanted horses in 2005 (Table 5). All ten received calls in 2010. The number of calls the charities received increased from one to thirty calls in 2005 to five reporting 50 to 100 calls, three reporting 100 to 150 calls one reporting 200-250 calls and one reporting > 250 calls in 2010.

**Table 5:** Number of Welfare Groups receiving calls relating to abandoned, neglected or roaming horses, and the number of calls they received

2005	2006	2007	2008	2009	2010	No of Calls
2	1					1-5

		2	2			5-10

1	1	2	2	1		10-30

	1	2	3	2		30-50

	1		2	3	5	50-100

				1	3	100-150

						150-200

					1	200-250

				1	1	250

**3**	**4**	**6**	**9**	**8**	**10**	**TOTAL**

#### Group size

Four calls were made to welfare charities about groups of 30 to 50 unwanted, neglected or abandoned horses in 2005 (Table 6). In 2010, there were 114 calls relating to groups of one to five horses, 61 calls relating to groups of 5 to 15, 41 calls relating to groups of 15 to 30, 43 calls relating to groups of 30 to 50, 149 calls relating to groups of 50 to 100, 35 calls relating to groups of 100 to 150, 150 calls relating to groups of 200 to 250, and one call regarding a group of > 250 horses.

**Table 6:** Group size of horses in calls made to welfare charities

HORSES	2005	2006	2007	2008	2009	2010
1-5	1	1	8	11	54	114

5-15	2	2	10	11	56	61

15-30	3	3	3	5	47	41

30-50	4	4	4	5	5	43

50-100				35	165	149

100-150					1	35

150-200						

200-250						150

> 250						1

#### Visits

In 2005, one of the ten welfare groups (Table 1) reported five to 10 visits and one reported 10 to 30 visits to unwanted horses (Table 7). In 2010 one group reported five to 10 visits, two reported 30 to 50 visits, two reported 100 to 150 visits, one reported 150 to 200 visits and a further two welfare groups reported 200 to 250 visits.

**Table 7:** Number of visits by welfare groups to abandoned, neglected or roaming horses

VISITS	2005	2006	2007	2008	2009	2010
1-5				1	1	

5-10	1	1	1	1		1

10-30	1			2		

30-50		1	2	1	3	2

50-100				2	1	

100-150					2	2

150-200						1

200-250						2

Age breed and gender data was provided by one welfare group on a total of 226 horses and ponies that it received calls about or visited from 2005 to 2010. 74 (33%) were < 1 year old, 55 (24%) were 1-5 years old, 38 (17%) were aged 5-10, 44 (19%) were 10-20, 11 (5%) were 15-20 and 4 (2%) were > 20 years of age. The same group reported gender categories on 248 horses they received calls about or visited of which 146 (59%) were female, 68 (27%) were males and 34 (14%) were geldings. They also reported breed categories on 227 horses and ponies they received calls about or visited between 2005 and 2010 of which 92 (40%) were Sport horses, 87 (38%) were ponies and 48 (21%) were thoroughbreds. They made no returns under the "No breed" category.

#### Horses found dead or requiring immediate or subsequent euthanasia

In 2005, nine horses were reported to have been found dead or required subsequent euthanasia on visits by welfare groups in (Table 8). In 2010, a total of 279 horses were reported either found dead or required immediate or subsequent euthanasia.

**Table 8:** Numbers of horses reported by Welfare groups found dead or requiring immediate or subsequent euthanasia

	2005	2006	2007	2008	2009	2010
Found Dead	2	1	10	13	72	137

Immediate Euthanasia			3	4	37	79

Subsequent Euthanasia	7	8	15	16	29	63

TOTAL			**21**	**33**	**138**	**279**

### Local authorities

#### Calls

In 2005 three of the seven Local Authorities (Table 1) reported two to five calls relating to unwanted horses, two reported five to 10 calls and another reported 10 to 20 calls (Table 9). In 2010, six of the seven Local Authorities reported calls relating to unwanted horses, one reported five to 10 calls, three reported 20 to 50 calls and two reported 50 to 100 calls.

**Table 9:** Number of Local Authorities receiving calls relating to abandoned, neglected or roaming horses and the number of calls they received

2005	2006	2007	2008	2009	2010	Calls
	1					**1 - 2**

3	2	5	3	1		**2-5**

2	2		1	3	1	**5-10**

1	1	1	2			**10-20**

				3	3	**20-50**

					2	**50 - 100**

**6**	**6**	**6**	**6**	**7**	**6**	**TOTAL**

#### Group size

In 2005, Local Authorities reported receiving a total of 32 calls that related to groups of one to five horses (Table 10). In 2010 Local Authorities reported receiving 136 calls relating to groups of one to five horses, 23 calls relating to 5 to 15 horses and 35 relating to 15 to 30 horses.

**Table 10:** Calls made to Local Authorities--group size

Horses	2005	2006	2007	2008	2009	2010
**1-5**	32	27	28	26	82	136

**5-15**					13	23

**15-30**						35

#### Visits

In 2005, three of the Local Authorities (Table 1) reported making a total of 28 visits that related to groups of one to five horses (Table 11). In 2010, five of the seven Local Authorities reported making a total of 86 visits to groups of 1 to 5 horses, 17 visits to groups of 5 to 15 horses, and 36 to groups of 15 to 30 horses and 158 visits to groups of 50 to 100 horses.

**Table 11:** Visits by local authorities-group size

Horses	2005	2006	2007	2008	2009	2010
**1-5**	28	21	21	19	42	86

**5-15**					18	17

**15-30**					1	36

**30-50**					48	

**50-100**						158

#### Seized and released

In 2005 six of the seven Local Authorities (Table 1) reported seizing a total of 38 horses and 4 Local Authorities reported releasing 30 (79%) of them (Table 12). In 2010, the seven Local Authorities reported making a total of 144 seizures of which 6 Local Authorities reported releasing 49 (34%) of them.

**Table 12:** Numbers of horses seized and released by local authorities

Horses	2005	2006	2007	2008	2009	2010
Seized	38	45	40	29	58	144

Released	30	16	25	18	15	49

#### Horses found dead or requiring immediate or subsequent euthanasia

Two of the seven Local Authorities (Table 1) reported finding a total of two dead horses on their visits in 2005 (Table 13). In 2010, five of the seven Local Authorities reported a total of 58 horses found dead, or that required either immediate or subsequent euthanasia.

**Table 13:** Numbers of horses reported by Local Authorities found dead or requiring immediate or subsequent euthanasia

Horses	2005	2006	2007	2008	2009	2010
Found Dead	2	3	2	5	11	18

Immediate Euthanasia					1	3

Subsequent Euthanasia				1	2	37

TOTAL	2	3	2	6	14	58

#### Returns to central government by all 35 Local Authorities

Local Authorities seized 714 horses in 2005 and 2,364 horses in 2010. Total seizures for 2005 to 2010 were 7,312 (Table 14). The categories of recording of local authority data on the disposal of horses they seized was changed in 2008, when horses returned to their owners was recorded, and in 2009 when horses returned to their owner or sold and those sent for slaughter were recorded. Local Authorities sent 589 horses to slaughter in 2010.

**Table 14:** Central Government Statistics on total national Local Authority seizures, releases and euthanasia of unwanted horses

	2005	2006	2007	2008	2009	2010	TOTAL
Seized	714	762	908	1,099	1,465	2,364	7,312

Disposed	208	191	246	385	n/a	n/a	1,030

Returned/Sold	n/a	n/a	n/a	n/a	443	643	1,086

Returned to Owner	n/a	n/a	n/a	10	n/a	n/a	10

Slaughter					21	589	610

(n/a--not applicable. The method of recording was re-classified from 2008 onwards)

**Figure 2:**
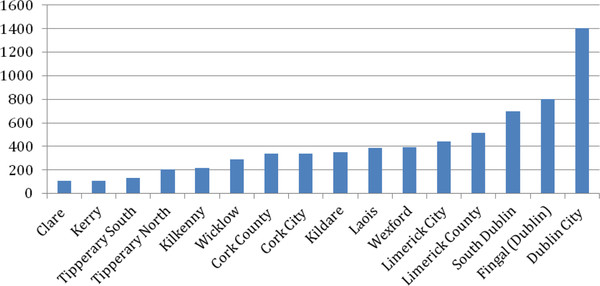
**Local Authorities reporting total seizures of > 100 unwanted horses from 2005-2010**.

16 of the 35 local authorities reported seizures of > 100 horses to central government from 2005 to 2010 (Figure 2). 2,906 of the 7,312 seizures (39.7%) were reported by Dublin City, Fingal and South Dublin and 954 by Limerick City and County (13%). Cork City and Cork County reported a total of 678 seizures (9.2%). Total seizures for the general urban and surrounding areas of Dublin, Limerick and Cork were 4,538 (62.1%).

### Category 2 plants

#### Numbers and age

The six Category 2 plants (Table 1) reported a total of 551 horses processed in 2005, 785 in 2010 and the majority of these horses were aged from < 5 to 15 years of age (Table 15). 100% of the horses processed by two of the six Category 2 plants had no passports; 50% of the horses processed by another Category 2 plant had no passports.

**Table 15:** Number of horses processed at Category 2 plants and their ages

	2005	2006	2007	2008	2009	2010	TOTAL
Total processed	551	520	582	661	712	785	3,811

Age (years)							

< 5	12	8	15	10	20	27	

5-10	37	36	40	42	38	53	

10-15	10	5	12	13	13	49	

15-20	7	9	8		7	5	

> 20	5	3	5		5	5	

#### Gender and type

A total of 374 of the horses reported to have been processed at Category 2 plants (Table 1) had gender reports of which 266 (71%) were females and 108 (28%) were entire or castrated males (Table 16). 402 were classified by type of which 267 (66%) were classified as ponies and 135 (33.5%) as "Thoroughbred".

**Table 16:** Category 2 plants-gender and "type" of horses

	2005	2006	2007	2008	2009	2010	TOTALS
Male or Gelding	13	10	15	14	14	42	108

Female	38	36	45	37	54	56	266

Pony	24	19	45	35	54	90	267

T'bred	20	20	17	18	20	20	135

Sporthorse	0	0	0	0	0	0	

No breed	10	10	8	2	0	0	

### Abattoirs

There are three DAFF approved abattoirs in the Republic of Ireland. There are also two Local Authority approved abattoirs, one in Limerick and one in Offaly. Only one of the abattoirs responded to the survey. This abattoir had begun slaughtering horses in 2010 and slaughtered 110 horses in that year. All horses had passports and 80 also had microchips.

The total numbers slaughtered at the three DAFF abattoirs rose from 783 in 2005 to 7,296 in 2010 and the total for the study period was 15,629 (Table 17).

**Table 17:** Numbers of horses slaughtered at DAFF Abattoirs

	2005	2006	2007	2008	2009	2010	TOTAL
Horses	783	822	1,506	2,002	3,220	7,296	15,629

**Figure 3:**
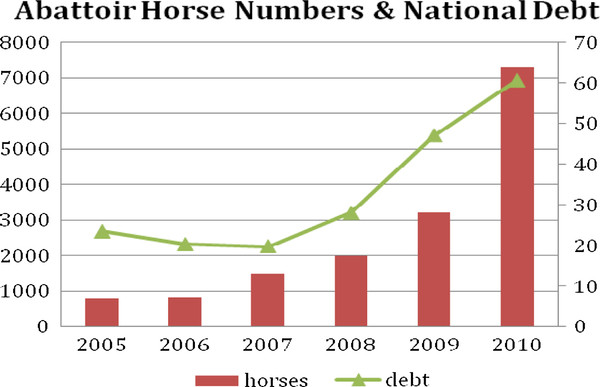
**Total Horse numbers slaughtered at the three DAFF approved abattoirs and Total Central Government Debt% of GDP **[16].

The total numbers slaughtered at these three DAFF approved plants was graphed (Figure 3) in relation to Ireland's Total Central Government Debt expressed as a percentage of Gross Domestic Product (GDP). The number of horses slaughtered increased concurrently with this measure of national debt.

Trichinella testing at the Irish Equine Centre began in 2005 with one abattoir, increased to three abattoirs in 2008 and to four in total in 2010 (Table 18). (The fifth abattoir commenced sample submissions in 2011). Peak submission of samples for equine Trichinella testing in 2010 occurred in September, October and November (Table 19).

**Table 18:** Annual totals of Irish Equine Centre sample receipts for equine Trichinella testing data

Year	2005	2006	2007	2008	2009	2010
**Samples**	493	837	1,497	1,878	3,256	8,556

**Table 19:** Monthly totals of horses slaughtered for human consumption at all five Irish abattoirs January to December 2010-based on IEC samples for equine Trichinella testing data

Month	Total
January	193

February	549

March	424

April	406

May	503

June	483

July	537

August	736

September	1,252

October	1,550

November	1,270

December	653

**TOTAL**	8,556

FAO statistics on numbers of horses slaughtered in the EU are available up to and including 2009. Figures for 2010 are not yet available. A total of 314,513 horses were slaughtered in the EU in 2009 and the EU total for 2005 to 2010 was 1,575,755 (Table 20). Italy is the country with the largest EU slaughter horse total 97,648 in 2009 and 596,227 (37.9%) for 2005 to 2009. Ireland's total for these FAO statistics for 2005 to 2010 was 33,800 (2.1%).

**Table 20:** Total horses slaughtered in the EU 2002-2009 (FAO Statistics)

Country	2005	2006	2007	2008	2009	Total	%
Italy	136,213	165,797	98,921	97,648	97,648	596,227	37.9

Romania	50,000	55,000	52,000	52,000	53,000	262,000	16.6

Poland	33,239	33,508	39,608	33,166	40,031	179,552	18.0

Spain	24,929	26,100	24,900	31,000	32,000	138,929	8.8

UK	20,000	21,000	20,000	20,000	20,000	101,000	6.4

France	21,631	19,467	17,800	16,200	15,500	90,598	5.7

Greece	15,000	15,500	15,000	15,000	15,000	75,500	4.8

Germany	9,866	9,570	9,726	9,498	9,315	47,975	3.0

Belgium	11,039	10,460	10,466	9,209	9,000	50,174	3.2

Ireland	8,000	6,600	6,400	6,400	6,400	33,800	2.1

**TOTALS**	331,922	365,008	296,828	292,129	299,903	1,575,755	

## Discussion

The ITBA and the affiliates of HSI represent the high value regulated sector of Ireland's horse population. None of these owner groups had a recording system similar to the present survey in place hitherto which may have been a deterrent to respond. Although 287 responses from individual owners in the high value sector were received they represented only a small proportion of the total sought and we can draw no inference from the 98.7% non-respondents. However, the owner respondents had in excess of 2,500 horses in their care, in each of the years surveyed. Survey of individual high value horse owners may not initially appear to be a useful method of gathering data on unwanted horses, but it may, as in this case, reflect percentage usage of the various means of disposal that are available. Sale of horses through public auction, private sale and sale to dealers are the primary means of trade-related disposal. The percentage of high value horses that were retired, re-homed or euthanised exceeded those sent to Category 2 plants or abattoirs. Fewer high value horses were disposed of through category 2 plants and abattoirs than by any other means. There was an increasing demand for re-homing over the survey period. High value horse owning respondents sent a total of more than 400 horses for re-homing from 2005 to 2010. Re-homing capacity is limited and as seen in the USA, demand can rapidly exceed the welfare societies' ability to provide it.

Survey of the low value non-regulated sector was beyond the resources of the present study. A significant number of owners of low value horses may not have access to computers or email. Our percentage response results for the high value sector suggest that owners of low value unregulated horses, may therefore be even less responsive and so email surveys of them are likely to be unproductive. Other means of assessing their contribution to the unwanted horse population are required.

The majority of horses euthanised by private veterinary practitioners are euthanised on medical grounds and only a small percentage of total horse euthanasia by veterinarians is at the request of welfare groups, local authorities or the Gardai. However, the total has been increasing, as have the number of veterinary visits made and the size of the groups of horses involved, reflecting the increasing involvement of the profession in the care of unwanted horses.

Welfare societies stated that their limited resources have not included provision for detailed recording or providing personnel or finance for it. This survey has provided a template, which lends itself to further data gathering on an annual basis and welfare groups in particular should be encouraged to actively participate in this process henceforth. In addition, guidelines for the operation of equine sanctuaries already exist and should be utilized.

The number of calls to Welfare Societies increased throughout the survey period and more than doubled each year from 2008 onwards. Large groups of horses generated large numbers of calls to these groups. While some charities made small numbers of visits to abandoned, neglected or unwanted horses, others made up to 250 visits and the visit totals for 2009 and 2010 were much higher than in previous years. The figure of 2,014 calls made to one welfare group and the increasing numbers of calls and visits reflect the growing problem and the level of public concern. The majority of calls and visits related to young horses (57% were < 5 years of age), females (59%) and 78% were either sporthorses or ponies. The numbers of horses per visit again increased from 2008 to 2010. It is clear that welfare groups, which have limited resources, are providing a very important service to unwanted horses and to the community. Their participation to this survey has helped to quantify the extent of the unwanted horse problem in this country.

Unsubstantiated claims of the extent of the problem, such as the New York Times report of "tens of thousands of horses reported abandoned" have been very damaging to the Irish horse industry (the Thoroughbred sector alone provides employment for > 22,000 people) and to Ireland's international reputation.

The combined total number of horses found dead or requiring immediate or subsequent euthanasia by welfare groups and local authorities in 2010, the majority of which are by definition, low value horses, was 337 and this must be a major national concern. Furthermore, although the percentage of these horses either found dead or requiring immediate or subsequent euthanasia represented a relatively small percentage of the total horses visited by these groups, mortality is a poor indicator of the degree of neglect. Objective recording of condition/body score would be of benefit to these societies and to society as a whole.

Calls to Local Authority Offices in relation to abandoned neglected or roaming horses increased significantly in 2009 and 2010 as did the group sizes. The numbers seized increased throughout the study period. More horses entered the care of and/or are euthanised by Local Authorities than the Welfare Societies, (although Local Authorities may be alerted to some of these horses by the Welfare Societies who cannot provide the service themselves). Local Authorities seized a total of 7,312 horses and sent 610 to slaughter. Local Authority involvement in unwanted horses represents a very substantial degree of tax payer/state investment in personnel resources and expenditure. The seized to released ratio in the single Local Authority response differed from the national rate, where the percentage released was much higher. Here as with the welfare societies, in the future, an objective measurement of condition/body score of horses entering their care would be of value.

Local Authorities, Category 2 plants and Horse Abattoirs all participate in central government statistical compilation and these data are freely available. However the demographic information on horses in each of these three categories is limited and could be extended to enhance understanding, and local and national policy and legislative decision making. The importance of enhanced demographic data is shown in that more females than males and more ponies than horses were reported from one Category 2 plant. This finding is in contrast with the type and sex distribution in the US demographic study of equine rescue and sanctuary organizations, once again illustrating the need for improved demographic data in Ireland.

The vast majority of unwanted horses in Ireland are disposed of through the Category 2 plants and the abattoirs. There has been a progressive increase in disposal through both of these systems throughout the economic downturn. This has been concurrent with well documented decreased production in the elite horse sector and increased national indebtedness. Whether decreased production has also occurred in the unregulated sector is not known, nor is it known whether the figures for Category 2 plant and abattoir disposal represent a peak and will be followed by decline, mirroring a decline in the total Irish Horse population, in addition to the well documented decline in the thoroughbred and sport horse sectors. Licensing, control and inspection of Category 2 plants and horse abattoirs also involves commitment of state and taxpayer resources. It is clear however, that there is an ongoing need for these very important, efficient and humane means of disposal and that their absence or closure, as in the USA, can rapidly create major welfare and social issues.

All of the horses presented to two of the Category 2 plants had no passport, and 50% of those presented to a third had no passport. As these horses would not have been suitable for the food chain the responsible owners paid to have them humanely destroyed and the carcasses rendered. However other owners may be unwilling or unable to pay the costs for disposing of horses that are not eligible to enter the human food chain.

The number of horses disposed of by owners by sale to a dealer increased. While these sales represent the traditional trading practices of the industry and in particular the sport horse sector, to some this may represent a means of disposing of unwanted horses at the least cost.

This may be a point of concern in that a large number of horses, who may not have good documentation, leave responsible ownership and become untraceable. Additionally, owners who responded to this survey represent the more regulated parts of the industry. The extent of this problem in the unregulated sectors cannot be ascertained from this data. The root problem is lack of traceability within the equine industry which is being addressed. Although owners are legally obliged to hold passports for horses, this is not stringently enforced and there is an unknown quantity of horses without passports. Additionally, even where passports are obtained, keepers are not legally obliged to update ownership details when horses change hands and there is no central database for horses registered with the passport issuing agencies other than for the Thoroughbreds and Sportshorses in the country.

The number of horses sent for slaughter in Ireland has increased dramatically. Stud farmers, racing trainers and many other keepers of horses on behalf of others, have been reporting being left with horses abandoned by their owners who can no longer afford to pay for their keep and who no longer have any wish to have any involvement with them. The associated debts and ongoing maintenance costs mean that there is literally no alternative to slaughter in many cases.

The number of horses sent for slaughter in the EU has been declining and although FAO Statistics are not available throughout the study period, those from 2005 to 2009 indicate that Ireland produced a small percentage of the European total. FAO statistics are often estimates and the figure for the UK in 2010 of 7,993 and the figures for Ireland throughout the study period indicate that although they may have value in terms of relativity, they are not absolute in many cases. Investigation of horses sent outside this country to slaughter was beyond the scope of the present study. Although the numbers of horses slaughtered in Ireland have increased, the FAO data despite its limitations, suggest it is likely that Ireland remains a minority contributor to EU horse slaughter numbers. Other EU countries have a tradition of eating horse meat and the absence of this tradition in Ireland does not mean that we, or other countries without this tradition, can fail to respect those who do.

The Irish Equine Centre Trichinella data corroborated the DAFF abattoir totals in 2010 and as it includes submissions from the two non-DAFF Abattoirs, (which were not responsive to the survey and whose data may be commercially sensitive), the total of 8,556 probably represents the true total for 2010. It is salutary to note that the submissions to date (21^st ^October) for 2011 are already in excess of 12,000 samples, emphasising that the present crisis has not yet begun to resolve.

## Conclusions

The economic recession has resulted in a significant increase in the numbers of unwanted horses in Ireland. There are deficits in the currently available demographic data which need to be addressed and there is a need for annual demographic data compilation and review to assist all stakeholders, including industry, veterinarians, welfare groups, policy makers and legislators in the ongoing and future management of unwanted horses and ponies.

## Competing interests

The authors declare that they have no competing interests.

## Authors' contributions

DL initiated the study, obtained the funding, provided the basic format of the study, wrote the first draft of the manuscript and acted as study team leader. DO'T helped to create lists of survey participants and assisted in the collation of the data. VD participated in the design of the study, designed the on-line surveys, collated the data and was the equal partner and co-worker with DL on all subsequent drafts and on the final draft of the manuscript. All authors read and approved the final manuscript.
